# Engaging With Farmers to Explore Correlates of Bovine Tuberculosis Risk in an Internationally Important Heritage Landscape: The Burren, in the West of Ireland

**DOI:** 10.3389/fvets.2022.791661

**Published:** 2022-02-15

**Authors:** AnneMarie Clarke, Andrew W. Byrne, James Maher, Eoin Ryan, Fidelma Farrell, Catherine McSweeney, Damien Barrett

**Affiliations:** Department of Agriculture, Food and the Marine, Dublin, Ireland

**Keywords:** mycobacteria, biosecurity, farming for conservation, Ireland, bovine tuberculosis (bTB), survey

## Abstract

Bovith recene tuberculosis (bTB) continues to be a pathogen of concern in several countries globally. Analysis of areas that have higher incidences of bTB outbreaks has demonstrated how risk is not equally distributed, and local data collection, analysis and participatory engagement is required to develop tailored approaches. The Burren, an internationally important heritage landscape, has been an area of higher bTB incidence for many years in Ireland, and owing to its unique geology and farming heritage a survey was developed to engage with local farmers to gain greater insight into farming practices and bTB control to inform tailored approaches. The survey gathered data on the farm and animal management approaches being used within the Burren, including local farming techniques like the use of “winterage” (grazing exposed limestone dominated uplands). Thematic analysis of free text responses was undertaken. Quantitative data were then explored using statistical models to assess associations with recent (<3 years) self-reported bTB breakdown risk. There was a high number of responses demonstrating a high degree of willingness to engage on the issue. Thematic analysis suggested that wildlife and its management (culling and vaccination), testing quality, and its impact on the bTB scheme, and pessimism around eradication were important themes. Statistical analysis suggested that increasing bTB risk was primarily related to increasing herd-size and the percentage of herd owner's land inaccessible to those attempting to locate badger setts. There was less evidence for associations relating to the amount of time, or which season (i.e., summer), farmers utilized “winterage”. The results of the study will feed back directly to local bTB management plans and further stakeholder engagement and is an exemplar for local tailoring of national control measures in situations of high incidences of bTB outbreaks in particular areas.

## Introduction

Bovine tuberculosis (bTB) is caused by *Mycobacterium bovis*, with advanced infection resulting in chronic respiratory disease characterized by granulomas in affected tissues. This disease is a persistent problem having significant animal health and welfare consequences for cattle industries (1) and posing a risk to trade in cattle and bovine animal products. Eradication programmes are compulsory within the European Union (2) and this requirement has had economic consequences also as countries strive to achieve the “officially tuberculosis free” status. The costs of the bTB eradication programme have been significant for many countries, with direct sectoral costs of €98 m in Ireland in 2020 (3). Stakeholder involvement is a key element of the bTB eradication programme in Ireland, with the Irish bTB eradication strategy supported by a stakeholders TB Forum which meets regularly to input into policy development. Underpinning the development of bTB risk communication policy is an emphasis on engaging with and listening to those affected, so as to more effectively convey ways to reduce bTB risk. In Ireland there has been a long term reduction in the levels of bTB reported over the years, however this decreasing trend is not uniform across the country and improvements achieved were highly heterogeneous (4). The levels of chronic bTB infection can differ between local areas and high risk areas can be identified using a range of hotspot analysis such as those described by Milne et al. (5).

One of those high risk areas is the Burren which is located in County Clare in the mid-west of Ireland. County Clare is a known bTB area of higher incidence at herd and individual cattle level, with the north of the county including the Burren consistently have greater than national average risk (4). The epidemiology of bTB is entwined by the presence of wildlife hosts, for example the European badger *Meles meles* is a reservoir host of infection known to spillback infection to cattle (2). During 2009–2012, County Clare had the highest apparent prevalence of bTB positive badgers across counties detected from surveillance activities at 20% positive (95%CI: 15.9–24.4%) from 361 badgers sampled (6). Other wildlife are present in the Burren, including deer species and feral goat populations, though currently there are no data supporting their involvement in bTB epidemiology in the Burren. There is some evidence of local involvement of deer in bTB epidemiology in high density areas of Wicklow in the east of the county; however this is a very different ecological area from the Burren (7).

The Burren is an internationally important heritage landscape, owing to its unique geology and farming heritage with ancient agricultural practices dating back for 6,000 years ago (8). There are a number of farming practices unique to livestock farming in the Burren that warrant further investigation in the context of the epidemiology of the bTB. For example, there is an emphasis on farming for conservation and use of traditional practices of sharing of land parcels (commonages) and grazing of stock on “winterage”. The objectives of this study were to investigate whether particular farming practices in the Burren were associated with recent bTB outbreak risk and obtain knowledge on the farmer attitudes to bTB outbreaks in this region, with a view to informing policy development in relation to more effective risk communication to farmers.

## Methodology

### Study Location

The Burren is located in County Clare in the mid-west of Ireland and is characterized by large amounts of bare rock, thin soils and patchy vegetation (9). This area encompasses in excess of 300 km^2^ and is recognized internationally for its karst geology and unique flora (10). Traditionally, sheep and cattle are the most popular animals that graze on the Burren. Farmers also kept goats for milk supply but there are few farmers who now actually herd goats. However, the progeny of these goats is still seen today and roam free across the Burren as feral or “wild” goats. Traditional farming methods and community farming practices are commonly practiced on the Burren, for example, commonages where farmers share rights to land parcels for grazing are a common feature of the upland areas of the Burren. In the Burren, it is common for livestock graze the uplands (winterages) in winter and the lowlands in summer, a practice that has been referred to as “reverse transhumance” (11). Reasons for the use of this winterage include that the lowlands of the Burren are often flooded during the winter (due to the porous nature of limestone pavement) and water availability is limited in the uplands during the summer. Also, the limestone rock absorbs heat during the summer and retains this heat during the winter resulting in more grass growth during colder times in comparison to other upland areas.

### Study Design

A self-completion survey using the *SurveyMonkey* software package was created by the OHSS team in DAFM, with expertise in social science, veterinary, epidemiology and animal science. Input was also provided by veterinarians with responsibility for bTB eradication both nationally and locally in the study area. In total, there were twenty questions to be answered. An information section was presented at the outset, assuring potential participants that their anonymity would be protected and that their participation was voluntary, and the first question then asked respondents if they consented to participate in the study and to confirm that they were over 18 years of age. Eighteen quantitative questions, comprising multiple choice and ranking style questions, were included in the survey. Where multiple responses were available the order of the options was randomized to minimize responder bias. The exception to this was where there was a logical sequence in the potential answers, for example sizes of farm (question 3) or number of cattle (question 8), the options were grouped numerically in order of size. In the ranking style question, (question 11), the participants had to rank each of the options; with 1 being what they felt the most important factor was. The method used for scoring these responses was to assign a reverse score, i.e., in a question with four options the most important factor, ranked by a farmer at number one, was given a score of four. The weighted average was then used to determine the rankings. It was made compulsory to provide a response to each question before one could progress to answering the next question. The final question was an open question, asking participants to “*provide any further comments they had regarding bTB in the Burren*”. Qualitative research used in open questions asks participants “to describe their experiences in ways that are meaningful to them” (12). Although the findings of such research cannot be generalized to other contexts, this method helps provide a greater understanding of certain issues, through the unique perspective of the participants. The rationale for incorporating a qualitative component to this survey was to help improve DAFM's understanding of farmers' opinions on this topic and to allow participants to provide further information which they deemed important.

### Data Collection

A link to the survey was issued by SMS text message to all 520 farmers in the Burren region on January 4th 2021; all had voluntarily provided their mobile phone numbers to DAFM for the purposes of communication, including by SMS. 320 of these farmers, who are members of the Burren Life European Innovation Partnership scheme (13), also received an email containing the survey link. Two SMS reminders were subsequently issued on January 11th and 18th. By the closing date of January 25th, 300 respondents consented to participate in the study. This would suggest a response rate of approximately 57%, but it is not possible to determine an accurate response rate given the possibility that recipients of the text message may have forwarded it to friends and family to complete.

### Ethics

Research ethics protocols were followed. All project proposals for the One Health Scientific Support (OHSS) team within the Irish Department of Agriculture, Food and the Marine (DAFM), are approved through a Departmental governance and oversight process. Informed consent was achieved by providing an information section at the beginning of the survey. This section explained the aims of the study, assured participants that their participation was voluntary and that their confidentiality would be protected. Respondents were free to choose whether to consent to participate in the study by selecting the option in the first question. Quotes from the qualitative data are presented anonymously in this manuscript.

### Descriptive Analysis

SurveyMonkey was used to generate rating scales for the ranking style question, as in previous research including Sayers et al. (14). Data from the output file was then used to undertake statistical analysis (see section Statistical Analysis). Each of the questions and options for responses are presented in Supplementary Material (Supplementary Table S1). Due to small numbers of responses in some questions, due to participants dropping out, some categories were grouped in order to allow for further analysis. The final dataset used in the analysis is presented in Supplementary Table S2.

The twentieth and final question of the survey asked participants to “provide any further comments you have regarding bovine TB in the Burren”. One hundred and sixty participants who had completed all the other quantitative questions ended the survey without providing any further comments. A total of 110 responses were received for this question (representing 37% of the respondents who had consented to participate in the study). The first step in the analysis of these free text responses was to identify any unusable replies, for example, where the respondent had indicated that they had no further comment. Six responses were removed at this stage, leaving 104 substantive responses for further analysis. This question produced a significant volume of qualitative data, coming to a total word count of 4,764 words.

Each of the individual response was read and assigned a tag, or multiple tags where appropriate, categorizing the responses as falling under specific themes. It is not claimed that the list of themes used to categorize the qualitative responses is an exhaustive one, rather the themes cover those topics which could be clearly defined and which arose in multiple responses. Twenty six of the responses to this question referred, in whole or partially, to issues that could not be categorized clearly within these chosen themes. Certain select quotes were be used to illustrate some of these miscellaneous topics in this paper.

### Statistical Analysis

In order to explore correlates of bTB risk, all herds were classified as either being recent bTB positive or negative, based on self-reporting from participants. All herds were classified as “positive” if herd owners reported that their herds experienced as bTB breakdown within the last 3 years. All other herds were classified as “negative” for recent bTB breakdown.

All categorical variables allowed for only one category per herd. Where a farmer selected two options for herd type, only one was used in the model. The response was always taken as the option with the subjective higher bTB risk, given previous research from Ireland [e.g., (2, 15–17)]. Explicitly, if a farmer picked two different herd types (i.e., a mixed herd), one including a dairy type herd, then that herd was given the indication of “dairy”. The two dairy herd types (breeds replacements and purchases replacements, respectively) were also merged as there were few responses for model fitting.

Throughout, the binary outcome for models was used to assess whether there were any associations with putative risk factors, based on survey responses. Logit regression models were used to model these associations. All independent variables were categorical factor variables. Each putative risk factor was assessed for [unconditional] association with the outcome using univariable models. Variables were then offered to multivariable models, with model building completed in two ways. Firstly, parsimonious models were selected using automated backwards, forwards, and stepwise selection procedures (using Stata's STEPWISE function). In addition, as there were many independent variables and a relatively small dataset, a multi-model comparison was also undertaken using Akaike's Information Criteria, corrected for small sample size (AICc). The premise being that the “true” model may be one of a set of candidate models (18, 19). Each combination of factors was ranked by their AICc and reported. Models with the lowest AIC values were considered the preferred model. Furthermore, we report on the probability of inclusion statistic for each variable being included in competing all-possible models based on information criterion weights (19, 20). This inclusion probability for each independent variable is an indicator of “usefulness” of each respective variable, an indication of variable importance (20). No interactions were tested in these models.

## Results

### Association Between bTB Recent Risk and Farmer Reported Responses

Initially, 280 participants completed the first survey questions, meaning twenty participants dropped out immediately after consenting to take part (*n* = 303). The majority of farms in the Burren were classified as suckler farms (64%) (where beef cows are present, calve each year and where replacements are mostly bred on the farm). Other farm enterprises included suckler herds where replacements are purchased (19%) and herds where cattle were purchased and fattened for sale (14%). Less than 10% of farms were dairy enterprises.

Regarding the last bTB detection in a herd, the largest cohorts (35%; 97/280) of farmers either had their last bTB outbreak over 10 years ago or have never had a breakdown. Approximately 30% (83/280) of farmers had bTB detected in their herd within the past year, while a further 13% of Burren farmers last had bTB in their herd between 1 and 3 years ago (35/280). This meant for our modeling outcome, 118/280 (42%) herds were classified as a positive case (i.e., a self-reported bTB breakdown in the last 3 years).

#### Unconditional Associations

The unconditional associations with this outcome, when p<0.1, are presented in Table 1 (all univariable associations are presented in the Supplementary Material, Supplementary Tables S3–5). These models suggested that there were associations between the outcome and several independent variables. There was a trend toward increase in risk with increase herd size category, in herds with dairy enterprises relative to other herd types, and with the increase proportion of farm area that was inaccessible for those seeking to locate badger setts due to the presence of scrub/hazel habitats. There was some evidence for increasing risk for herds using winterage for ≤1 month in the winter season relative to herds that did not use winterage or used winterage for longer periods of time. Related to this, there was a trend toward greater risk to be associated with for summer grazing on winterage.

**Table 1:** Associations between survey responses and the self-reported status of herds in relation to bTB detection during the previous 3-years.

**Variable**	**Categories**	**N**	**N+**	**% +**	**OR**	***P***	**Lower 95%CI**	**Upper 95%CI**
Herd size		*280*	*118*	*42.14%*		*0.026*		
	0–30	109	34	31.19%	Ref			
	31–50	51	21	41.18%	1.544	0.217	0.775	3.076
	51–100	75	36	48.00%	2.036	0.022	1.108	3.739
	>100	45	27	60.00%	3.088	0.001	1.609	6.805
Badger sett inaccessibility		*270*	*112*	*41.48%*		*0.001*		
	<10% of the farm	181	65	35.91%	Ref			
	Between 11–30% of the farm	64	27	42.19%	1.302	0.373	0.727	2.329
	Between 31–50% of the farm	12	8	66.67%	3.569	0.044	1.034	12.309
	Over 50% of the farm	13	12	92.31%	21.41	0.004	2.722	168.441
Time on winterage		*274*	*113*	*41%*		*0.0417*		
	1 month	14	11	78.57%	Ref			
	2 months	26	9	34.62%	0.144	0.012	0.318	0.654
	3 months	69	31	44.93%	0.222	0.031	0.569	0.868
	4 months	87	33	37.93%	0.166	0.009	0.0432	0.641
	0 months	78	29	37.18%	0.161	0.008	0.0415	0.627
Summer grazing of winterage		*274*	*113*	*41%*		*0.074*		
	No	246	97	39%	Ref.			
	Yes	28	16	57%	2.048	0.076	0.929	4.517
Dairy		*276*	*116*	*42.03%*		*0.058*		
	No	249	100	40.16%	Ref.			
	Yes	27	16	59.26%	2.167	0.061	0.966	4.864

#### Multivariable Associations

The composition of the ten highest ranked models are presented in Table 2 below. The model with the lowest AICc included only two variables, “badger” (which represented the proportion of the farm area that was inaccessible to those seeking to locate badger setts,) and herd size. This model was also the model selected using backward, forwards and stepwise model building procedures. However, there was support based on the difference in AIC that the true model may also include “dairy” (ΔAICc = 0.863), “summer” (ΔAICc = 1.039), or both (ΔAICc = 1.758).

**Table 2:** Ranking of top 10 candidate multivariable models based on the Akaike's Information Criterion (AIC) corrected for small sample size.

**Model rank**	**Diff. AICc**	**Independent variables included**
1	0.000	badger + herd size
2	0.863	badger + herd size + dairy
3	1.039	badger + herd size + summer
4	1.758	badger + herd size + dairy + summer
5	2.709	badger + dairy
6	2.750	badger + herd size + winterage time
7	3.187	badger + dairy + summer
8	4.108	badger + herd size + dairy + winterage
9	4.585	badger
10	4.587	badger + herd size + summer + winterage

Based on the multi-model comparisons, the inclusion probability for each variable is presented in Table 3. The badger and herd size variables had a posterior inclusion probability of 0.996 and 0.812, respectively, dairy and summer had inclusion probabilities of only 0.451 and 0.374.

**Table 3:** Posterior inclusion probability of independent variables in the multi-model set.

**Variable**	**Inclusion probability**
Badger	0.996
Herd size	0.812
Dairy	0.451
Summer grazing	0.374
Winterage time	0.164

The top ranked model is presented in Table 4, and a comparison of parameter estimates across the top 10 ranking models presented in the Supplementary Material Table S6.

**Table 4:** Top ranked multivariable logistic regression model of associations between farmer survey responses and recent bTB breakdown risk in the Burren.

**Independent var**.		**Odds Ratio**	**z**	**P>z**	**Lower 95%CI**	**Upper 95%CI**
% land badger sett location inaccessible						
	≤ 10%	Ref.				
	11–30%	1.377	1.050	0.294	0.757	2.504
	31–50%	3.932	2.120	0.034	1.111	13.912
	>50%	19.705	2.810	0.005	2.466	157.432
Herd size						
≤30	Small	Ref.				
31–50	Medium	1.401	0.900	0.366	0.674	2.910
51–100	Large	2.172	2.380	0.017	1.146	4.118
>100	V. large	3.084	2.840	0.004	1.419	6.704
Constant		0.349	−4.400	0.000	0.218	0.558

The top ranked model suggested that herds where farmers reported that 31–50% or over 50% of their land was inaccessible for those seeking to locate badger setts had 3.93 (OR 95%ci: 1.11–13.91; *p* = 0.034) and 19.71 times the odds (OR 95%ci: 2.47–157.43; *p* = 0.005) of recently having a breaking down, respectively, relative to herds with 10% or less of their land inaccessible, while controlling for herd size.

**Figure 1:**
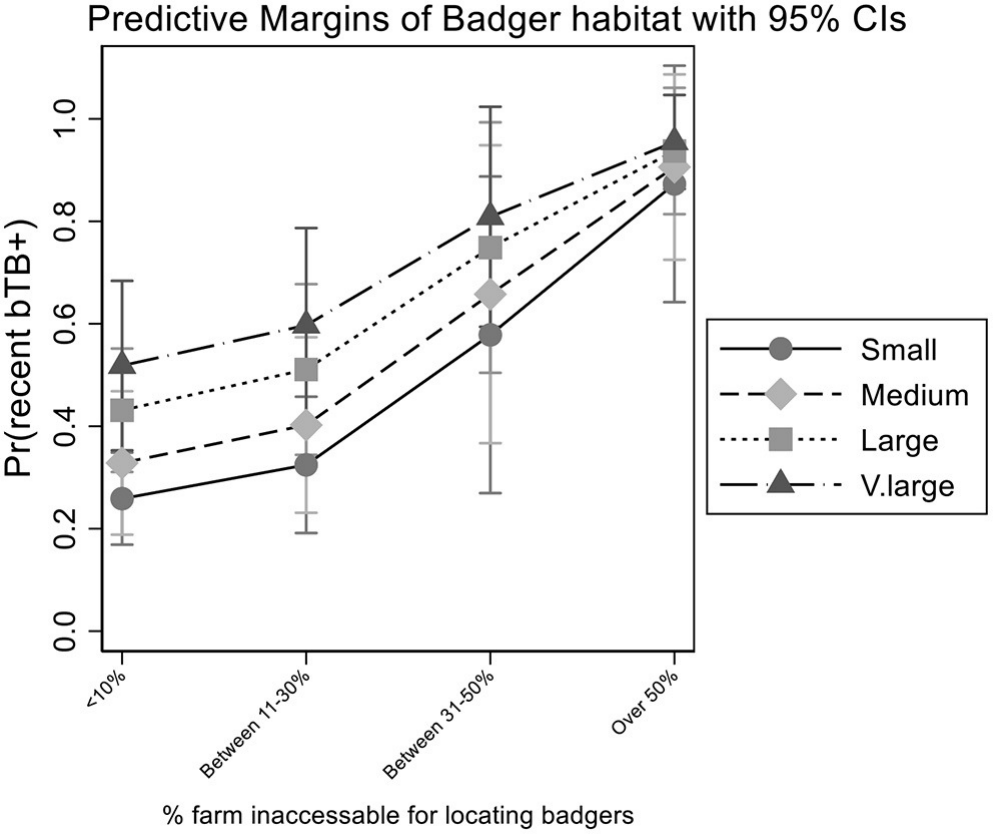
Relationship between the risk of recent bTB breakdown and herd size (small, medium, large and very large) and the percentage of farms that were reported accessible for locating badgers.

Large herds of 51–100 animals and very large herds of >100 animals were 2.17 (OR 95%ci: 1.15–4.12; *p* = 0.017) and 3.08 (OR 95%ci: 1.42–6.70; *p* = 0.004) times the odds of recently experiencing a breakdown, respectively, relative to herds with ≤30 animals. The predicted increase in risk by herd-size and badger sett inaccessibility percentage is presented in Figure 1. Very large herds where badger sett inaccessibility percentage is >50% are predicted to have experienced a high risk of bTB break down. Reciprocally, small herds where badger setts are easily located have low predicted probability of recently experiencing a bTB breakdown.

### Qualitative Results

This section aims to present the results of the final, open-ended question of the survey. This question gave respondents a chance to voice any further opinions they had on the topic of bTB in the Burren. The responses were categorized into themes and the main themes emerging from the analysis are presented here.

#### Wildlife

This was, by a large margin, the most common topic raised in the free text responses, with 61 responses referring to the role of wildlife in the Burren. Thirty four responses mentioned badgers and 33 responses mentioned feral goats. The majority of comments regarding badgers spoke about their role in spreading and perpetuating bTB; “*while badgers are there TB will remain*”. Many farmers spoke about the challenge of locating badgers, which can be especially difficult in the Burren; “*more work needs to be done on identifying sets [setts] and following up when badger activity is noted*”. Several respondents believed DAFM needed to place more emphasis on managing badger's role in bTB; “The *department of agriculture needs to go after the source of TB i.e., the badger with the same vigorous approach they go after the farmer and his cattle. It's disgraceful testing innocent cattle and badger continues to reinfect*”.

The responses regarding feral goats in the Burren could broadly be divided into two groups. The first group are convinced that the feral goats are one of the reasons for the high bTB incidence in the Burren; “*There is a massive population of wild goats in the area. The likelihood is that the vast majority is carrying TB. If the department is serious about eradicating TB they need to be culled immediately*.” The second group were unsure of feral goats' role in spreading bTB but hoped that research would be carried out to examine this possibility; “*Further research needs to be carried out and this should be done by testing a large sample size of goats in the Burren to examine if they have signs of infection*”. Many of the responses mentioning goats spoke about the practical difficulties they cause for farmers in the region by damaging boundary walls and fences, eating grass and meal.

Several farmers spoke about the need to shift emphasis from testing cattle to wildlife management of badger and feral goats; “*Same efforts should be put in to wildlife as the testing*”. Fourteen respondents mentioned culling these two wild species on the Burren, the majority of these farmers being in favor of a cull. Only two Burren farmers mentioned deer in their responses.

#### Testing

The second most common theme emerging from the thematic analysis related to bTB testing; which arose in 33 responses. The two main concerns Burren farmers had related to the frequency and the accuracy of the tests used to detect bTB. Many Burren farmers felt having several tests a year was excessive and a wasteful use of Departmental resources; “*I had three herd tests in 2020 one in the middle was done by the Department and all clear why was it deemed necessary for me to do last test as I see this as a waste of public funds*”. Several farmers suggested that an annual herd test would suffice; “*It is totally ridiculous to be constantly testing farmers who never have TB, it's so hard to manage testing in the Burren, if you are always free from TB you should not have any more than a once yearly test*”.

Many farmers expressed frustration at the inaccuracy of current bTB tests; “*The testing system is inadequate. It hasn't worked for the past 45 years and it won't work going forward*.” Several respondents believed the inaccurate testing leads to healthy animals being slaughtered as reactors; “*Farmers having good quality and healthy cows removed from their herds year on year and when they go to the factory they don't kill out with any TB. Could also end up having clear blood tests. There needs to be a more accurate system of identifying Tb carriers. It is time consuming and these 4 months testing is a very high cost on the Department every year with very poor results*.”

Some farmers spoke about the hardship for the farmer and animal welfare issues associated with bringing animals back off the winterage for testing, “*putting heavy in calf cows at high risk of losing their calf when they are being handled for testing*”.

#### Organizations

Sixteen Burren farmers mentioned DAFM in their responses. The majority of these responses referred to the Department needing to try new methods to reduce bTB. Farmers expressed frustration at the status quo, particularly the Department's rules on testing frequency; “*I do not understand how the Department expect by doing the same testing more often will bring a different result*”. Burren Life and Teagasc were each mentioned in one response. No other organizations were mentioned by Burren farmers.

#### Vaccine

Nine Burren farmers spoke about use of a vaccine to help eradicate bTB. The majority of these respondents spoke about the need for more widespread vaccination of badgers in order to prevent bTB spread; “*encourage vaccination of the badger, eliminate the chances of passing it on to the cattle*”. A small number of these farmers spoke about the need for further research leading to the development of an effective vaccine for cattle.

#### Eradication

Eight Burren farmers spoke about bTB eradication. The majority of these responses were pessimistic about the possibility of eradication being achieved based on current strategy, as seen in the following comment; “*The approach taken for TB to date will never eradicate the problem and the department should be clear on this*.” Four Burren farmers referred to the idea of bTB as an industry; “*this testing is a scam that farmers pay for. Have we a plan to end this once and for all*”.

#### Other

Biosecurity (*n* = 4), most of which mentioned the difficulty of maintaining boundary walls due to feral goats, water sources (*n* = 3), and cattle movement (*n* = 3) were the next most frequently mentioned topics.

## Discussion

The present study represents the first study of participatory epidemiology of bTB in an important heritage landscape in Ireland, utilizing both quantitative and qualitative approaches. The level of engagement with our survey suggested that farmer stakeholders are very keen to engage and share their views on the control of bTB in their local region. The responses allowed for additional local potential factors of interest from a bTB control perspective to be explored, including exploration of the utilization of “winterage” a unique farming approach to the Burren. The survey results allowed us to investigate associations with having recent bTB breakdowns (in the last 3 years) and several significant associations were identified. The results will inform improved policy development by DAFM, particularly in relation to bTB risk communication and stakeholder discussions on how best to engage with herdowners at the bTB Forum.

### Wildlife

Our statistical exploration of the survey data suggested that there was an association between recent bTB breakdown and the response to the question “what percentage of your entire farm is inaccessible (scrub or hazel) to locating badger setts?” There was a trend toward increasing risk of recent bTB breakdown and the percentage of farm inaccessible to setts due to habitat. At face value, this result could suggest that herds with more suitable habitat for badgers may be at greater risk for bTB breakdown; indeed, previous research has suggested that there is an association between metrics of badger density and breakdown risk (21). Inaccessible setts on farm would reduce the ability for wildlife interventions to occur, be they badger culling activities or vaccination. Reducing badger captures could conceivably reduce the effectiveness of bTB related intervention (22). As our results are reliant on farmer responses, it is possible that those farmers that have recently experienced a breakdown are more aware of badgers, and their habitats, on their farm, relative to farmers who have been tested clear of bTB for some years. Previous surveys have suggested that farmers can be quite accurate at knowing when setts are truly present on their farms (23), and there can be a differences in proportion of farmers reporting badger activity depending on the level of infection in the local area. Wildlife management was the most commonly raised topic by the respondents in the survey indicating that they are aware of the importance of wildlife in the epidemiology of bTB. Several respondents proposed further badger management, indicating a high level of awareness of the potential role of badgers in the transmission of bTB and the role of vaccination in badgers in reducing the transmission of bTB from badgers to cattle (16). Some respondents also raised the role of feral goats in the transmission of bTB, however previous studies of feral goats culled in 2017 from this area have failed to find evidence of bTB infection in the feral goat population (A. Johnson, personal communication). From a policy perspective, this illustrates the challenges of communicating findings which did not reveal a risk, in the face of stakeholder beliefs that feral goats are involved in bTB transmission.

### TB Eradication Programme and Farmers Attitudes to bTB

In addition to the management of wildlife, several respondents made comments on the overall management of the bTB eradication programme. Some voiced concerns regarding demands created by frequent testing of animals for bTB and this reflected fatigue with the eradication programme. This finding is similar to that of Robinson (23) where it was found that bTB as a disease is only one of many competing priorities facing farmers and their resources. Stresses associated with regulations, farm inspections, bad weather, market forces and paperwork can result in efforts to prevent the spread of bTB on farms becoming less of a priority in a farmer's daily life. This paper provides a valuable insight into farmer thinking and opinions on bTB, including the expressed sense of powerlessness and lack of control. In a survey of farmers from Northern Ireland, Hamilton et al. (24) found that farmers felt “utterly powerless and in an intractable position that promoted a sense of despair that paralyzed them and made new learning unappealing and seemingly pointless”. The findings were consistent with Enticott et al. (25) who found that farmers believe they are unable to do anything about bTB but are keen for the government to intervene to help control the spread of bTB. Previous research in the UK has indicated a lack of trust in government, particularly in counties with a longstanding high bTB incidence (26, 27). This also provides a useful context for recommendations from the bTB Forum that more advice on biosecurity is required; our findings indicate that securing stakeholder engagement at individual level may be challenging.

### Herd Size and Dairy

The size of cattle herds has consistently been a risk factor for bTB breakdowns in Ireland and elsewhere when using large national-scale datasets [e.g., (15, 16, 28, 29)], and is therefore unsurprising that a significant relationship was found in the present study. Herds >100 animals, which in the present study represent very large herds, had 19 times higher odds of recent breakdown relative to herds of <30 animals. Herd size can be a proxy for several other important herd characteristic which may relate to bTB risk, including the extent (area), the number of neighboring herds, and the intensity of farming being undertaken (30). The statutory test exhibits high but non-perfect specificity, with estimates suggesting that 1/5,000 tests being false positives [Sp: 99.98%; (31)]. Therefore, there is a small increased risk of false positive test disclosure in very large herds—though this should be less a problem in the Burren where the most frequent herd size category reported was <30 animals.

A recent study found that in addition to herd size, the number of parcels of land used can be associated with increased risk, but not metrics of intensity based on nitrogen loading, with breakdown duration (32). Herd size has been found to remain significant when controlling for herd type in other studies, which can be a confounder in this relationship. In the present study, herds with self-reported dairy enterprises were most likely to be of large (41%) or very large (48%) size. Dairy was found to be positively associated with increasing risk of recent bTB breakdown (OR: 1.67), though the association was non-significant when controlling for herd size and badger inaccessibility (Supplementary Table S1).

### Winterage

Grazing winterage habitats is a unique farming approach confined to the Burren region, and is a result of the unique geology of the region (8). The exposed limestone formations can act like a “heat storage” unit, which has allowed for a unique flora to exist there and the possibility of extended grazing periods in winter. Animals are moved from lower altitudes where soils are deeper and dominated by pasture, to the plateau where species rich swards dominate. At univariable level there was some indication of a potential association between recent bTB risk and amount of time winterage was used during the winter season, and also whether winterage was used in summer months. It appeared using winterage for short periods (less than a month) in winter, and using winterage in summer, may be associated with increased bTB risk, however this relationship appears weak and it could be a false-positive association. The mechanism underlying this association has not been established, especially as other management related factors like sharing commonage, supplementing feed, the usage of feed troughs were all non-significantly associated. The relationship, if real, could be potentially related to wildlife exposure, for example, there was an association between summer winterage grazing and badger sett inaccessibility. There was a higher proportion of farms summer grazing with larger areas inaccessible to locating badger setts. For example, only 8% of herds with <10% of land area badger sett inaccessible summer grazed winterage; 31% of herds with >50% inaccessible to badger setts grazed winterage during summer months. Until further data are gathered on this finding, we can only speculate as to its mechanism and significance.

### Methodological Issues

This is a relatively small sample size but there was a good response. Furthermore, it was representative of the population in the Burren, which was the subject of this study. The survey was anonymous which prevented us from verifying the herd's bTB infection history as herd identifier and location was not obtained in the survey. However, this was necessary for data protection purposes and may have encouraged the farmers to express their views more freely.

## Conclusions

We sought to analyse risk factors through participatory engagement with farmers and get their perspectives on bTB eradication in a specific region in Ireland. The level of engagement in the survey suggests that farmer stakeholders are very keen to be involved in and help with the control and eventual eradication of bTB in their area. The responses to the qualitative questions indicate concerns around wildlife management, fatigue with disease eradication programme, the quality of testing applied in the bTB programme and general pessimism around eradication of the disease. There was some of evidence of a mismatch between perception of risk by farmers, and actual risk relating to feral goats, given available data suggesting there is no M bovis circulating within that population. Statistical analysis suggested increasing bTB risk was primarily associated with increasing herd-size and the percentage of herd owner's land inaccessible to locating badger setts. The exploration of the utilization of “winterage”, a unique farming approach to the Burren, showed an indication of a potential association between recent bTB risk and amount of time winterage was used during the winter season and also whether winterage was used in summer months. Further research would be required to examine this latter finding in more detail. The results of the study can be utilized in local bTB management plans so that measures are tailored to the needs of this specific location.

## Data Availability Statement

The original contributions presented in the study are included in the article/Supplementary Material, further inquiries can be directed to the corresponding author.

## Author Contributions

AC drafted the manuscript. JM designed the study and administered the questionnaire. AB carried out the statistical analysis. FF and CM provided local input into the design and distribution of the survey. ER came up with the concept. DB oversaw the overall project. All authors have input into the production of the manuscript.

## Conflict of Interest

The authors declare that the research was conducted in the absence of any commercial or financial relationships that could be construed as a potential conflict of interest.

## Publisher's Note

All claims expressed in this article are solely those of the authors and do not necessarily represent those of their affiliated organizations, or those of the publisher, the editors and the reviewers. Any product that may be evaluated in this article, or claim that may be made by its manufacturer, is not guaranteed or endorsed by the publisher.
