# Supplementary material for: Engaging With Farmers to Explore Correlates of Bovine Tuberculosis Risk in an Internationally Important Heritage Landscape: The Burren, in the West of Ireland
Source: Front Vet Sci. 2022 Feb 15;9:791661. doi: 10.3389/fvets.2022.791661 (PMC8887599; doi:10.3389/fvets.2022.791661)
Supplement: Supplementary file 1 [file Supplementary_Material.docx]

**Supplementary Material**

**Table S1:** Survey questions and answer choices

1. Do you consent to participate in this study?

Yes; No.

1. When did you last have bovine TB in your herd?

Less than 1 year ago; Between 1-3 years ago; Between 3-6 years ago; Between 6-10 years ago; Over 10 years ago/never had a breakdown.

1. How many cattle (total cows, calves and all other age groups) do you currently have on your farm?

0-30; 31-50; 51-100; 101-150; 151-200; Over 200.

1. Please select all options that describe your farm type?

Suckler - breeds most/all of my replacements; Suckler - purchases most/all of my replacements; Dairy - breeds most/all of my replacements; Dairy - purchases most/all of my replacements; Purchases and fattens cattle for sale as store/beef; Other (please specify).

1. What proportion of your herd are put in winter housing?

Whole herd; More than half; Less than half; None.

1. How do you use winter housing?

To house younger cattle for full winter season; To house younger cattle for second half of winter; To house entire herd for full winter season; I don't use winter housing at all; To house adult herd for second half of winter; Other (please specify).

1. Do you use a winterage?

Yes; No.

1. How many cattle do you put on the winterage?

0; 1-25; 26-50; 51-75; 76-100; Over 100.

1. What is the size of the winterage you use?

1-50 acres (1-20 hectares); 51-100 acres (21-40 hectares); 101-150 acres (41-60 hectares); 151-200 acres (61-80 hectares); Over 200 acres (over 81 hectares); I don't use a winterage.

1. For how long do you have your cattle on the winterage?

1 month in winter; 2 months in winter; 3 to 4 months in winter; Over 4 months in winter; My cattle graze winterage during the summer; I don't use a winterage.

1. Please rank what is most important to you in deciding to bring your cattle off the winterage, with 1 being the most important. Please skip if you don't use a winterage.

Winterage has been grazed to ensure maximum payments under Environmental Schemes; Cows near calving; Sufficient feed for cattle not available; Cattle losing body condition.

1. Regarding the ownership/rental of rights to graze Burren commonage, which statement applies to you?

I do not own/rent commonage; I own/rent commonage but I do not graze it; I graze commonage with 1 other herd; I graze commonage with 2 to 3 other herds; I graze commonage with 4 or more other herds.

1. Do you feed silage to your cattle on the winterage?

I never feed silage; I only feed silage in bad weather events; I feed silage; I don’t put my cattle on winterage.

1. How many months do you feed concentrates when your animals are on winterage?

I don’t feed concentrates when my cattle are on winterage; 1 month; 2 months; 3 to 4 months; Entire winter period; I don’t put my cattle on the winterage.

1. Copper, Selenium and Iodine are important in an animal's diet. Deficiencies can occur throughout the year. Which statement applies to you?

I do not supplement cattle's diet with any trace minerals; I give Burren Concentrate feed (contains trace minerals); I give trace mineral boluses to cattle; I supply cattle with mineral licks; I give a regular commercial concentrate feed to cattle; Other (please specify).

1. How do you feed meal/concentrates to your cattle when they are on the winterage? Please select all options that apply.

In a raised trough; In a low trough (sits on the ground); Directly on the ground; I don’t feed any meal/concentrates when cattle on winterage; I don’t put my cattle on the winterage.

1. This is a question about the water supply for your cattle; please select all of the options that apply to your entire farm.

Cattle drink water from high troughs; Cattle drink water from low troughs; Water source is an open well/spring; Cattle drink water directly from streams/springs; Cattle can drink water from rock pools even when they have another water source; Water source is biosecure (for example - piped mains water, secure trapped spring or holding tank).

1. Liver fluke is a parasite that can occur on Irish farms. What treatment, if any, do you use for liver fluke?

I do not dose/inject my cattle for liver fluke; I treat my cattle once per year; I treat my cattle twice per year; I treat my cattle 3 times per year; I treat my cattle only when I suspect/know that they are infected.

1. What percentage of your entire farm is inaccessible (scrub or hazel) for locating badger setts?

Less than 10%; Between 11-30%; Between 31-50%; Over 50%.

1. Please feel free to provide any further comments you have regarding bovine TB in the Burren.

**Table S2:** Data set analysed in this paper

**Table S3:** Farm and herd characteristics associated with recent history of bTB breakdown on farm as reported from a survey of Burren farmers.

| Variable | Categories | N | N+ | % + | OR | P | Lower 95%CI | Upper 95%CI |
| --- | --- | --- | --- | --- | --- | --- | --- | --- |
| *Herd size* |  | *280* | *118* | *42.14%* |  | *0.026* |  |  |
|  | 0-30 | 109 | 34 | 31.19% | Ref. |  |  |  |
|  | 31-50 | 51 | 21 | 41.18% | 1.544 | 0.217 | 0.775 | 3.076 |
|  | 51-100 | 75 | 36 | 48.00% | 2.036 | 0.022 | 1.108 | 3.739 |
|  | >100 | 45 | 27 | 60.00% | 3.088 | 0.001 | 1.609 | 6.805 |
| *Winter housing prop.* |  | *278* | *116* | *41.73%* |  | *0.356* |  |  |
|  | < half | 52 | 20 | 38.46% | Ref. |  |  |  |
|  | > half | 51 | 27 | 52.94% | 1.800 | 0.142 | 0.822 | 3.942 |
|  | None | 100 | 40 | 40.00% | 1.066 | 0.854 | 0.537 | 2.121 |
|  | Whole herd | 75 | 29 | 38.67% | 1.009 | 0.981 | 0.488 | 2.086 |
| *Winter housing use* |  | *278* | *116* | *41.73%* |  | *0.211* |  |  |
|  | Don't use winter housing | 92 | 37 | 40.22% | Ref. |  |  |  |
|  | Other | 20 | 4 | 20.00% | 0.372 | 0.098 | 0.115 | 1.200 |
|  | Adult herd only | 44 | 19 | 43.18% | 1.129 | 0.742 | 0.545 | 2.339 |
|  | Entire herd | 58 | 23 | 39.66% | 0.976 | 0.945 | 0.499 | 1.911 |
|  | Young cattle full winter | 44 | 22 | 50.00% | 1.486 | 0.283 | 0.721 | 3.063 |
|  | Young cattle 2nd half winter | 20 | 11 | 55.00% | 1.816 | 0.23 | 0.685 | 4.814 |
| *Liver fluke treatment* |  | *270* | *112* | *41.48%* |  | *0.867* |  |  |
|  | Don’t treat | 31 | 11 | 35.48% | Ref. |  |  |  |
|  | Treat 3 times a year | 14 | 5 | 35.71% | 1.01 | 0.988 | 0.27 | 3772 |
|  | Treat once a year | 119 | 53 | 44.54% | 1.46 | 0.366 | 0.643 | 3.314 |
|  | Treat only when suspect infection | 29 | 11 | 37.93% | 1111 | 0.844 | 0.388 | 3.177 |
|  | Treat twice a year | 77 | 32 | 41.56% | 1.292 | 0.56 | 0.544 | 3.068 |
| *Badger sett inaccessibility* |  | *270* | *112* | *41.48%* |  | *0.001* |  |  |
|  | Less than 10% of the farm | 181 | 65 | 35.91% | Ref. |  |  |  |
|  | Between 11 - 30% of the farm | 64 | 27 | 42.19% | 1.302 | 0.373 | 0.727 | 2.329 |
|  | Between 31 - 50% of the farm | 12 | 8 | 66.67% | 3.569 | 0.044 | 1.034 | 12.309 |
|  | Over 50% of the farm | 13 | 12 | 92.31% | 21.41 | 0.004 | 2.722 | 168.441 |
| *Herd type* |  | *276* | *116* | *42.03%* |  | *0.124* |  |  |
|  | Purchases and fattens cattle for sale | 40 | 12 | 30.00% | Ref. |  |  |  |
|  | Suckler - breeds most/all of my replacements | 155 | 66 | 42.58% | 1.730 | 0.150 | 0.819 | 3.654 |
|  | Suckler - purchases most/all of my replacements | 54 | 22 | 40.74% | 1.604 | 0.285 | 0.674 | 3.818 |
|  | Dairy | 27 | 16 | 59.26% | 3.394 | 0.019 | 1.220 | 9.441 |
| *Dairy* |  | *276* | *116* | *42.03%* |  | *0.058* |  |  |
|  | No | 249 | 100 | 40.16% | Ref. |  |  |  |
|  | Yes | 27 | 16 | 59.26% | 2.167 | 0.061 | 0.966 | 4.864 |

**Table S4:** Land usage, including winterage, characteristics associated with recent history of bTB breakdown on farm as reported from a survey of Burren farmers.

| Variable | Categories | N | N+ | % + | OR | P | Lower 95%CI | Upper 95%CI |
| --- | --- | --- | --- | --- | --- | --- | --- | --- |
| *Use of Winterage* |  | 274 | 113 | *41.24%* |  | *0.252* |  |  |
|  | No | 78 | 29 | 37.18% | Ref. |  |  |  |
|  | Yes | 196 | 84 | 42.86% | 1.267 | 0.389 | 0.738 | 2.173 |
| *Winterage size* |  | *274* | *113* | *41%* |  | *0.359* |  |  |
|  | None | 78 | 29 | 37.18% | Ref. |  |  |  |
|  | 1 to 20ha | 49 | 16 | 32.65% | 0.819 | 0.604 | 0.385 | 1.739 |
|  | 21 to 40ha | 60 | 25 | 41.67% | 1.206 | 0.593 | 0.606 | 2.403 |
|  | 41 to 60ha | 34 | 18 | 52.94% | 1.901 | 0.122 | 0.841 | 4.294 |
|  | 61 to 80ha | 18 | 10 | 55.56% | 2.112 | 0.158 | 0.749 | 5.957 |
|  | Over 81ha | 35 | 15 | 42.86% | 1.267 | 0.567 | 0.563 | 2.853 |
| *Time on winterage* |  | *274* | *226* | *82%* |  | *0.0417* |  |  |
|  | 1 month | 14 | 11 | 78.57% | Ref. |  |  |  |
|  | 2 months | 26 | 9 | 34.62% | 0.144 | 0.012 | 0.318 | 0.654 |
|  | 3 months | 69 | 31 | 44.93% | 0.222 | 0.031 | 0.569 | 0.868 |
|  | 4 months | 87 | 33 | 37.93% | 0.166 | 0.009 | 0.0432 | 0.641 |
|  | 0 months | 78 | 29 | 37.18% | 0.161 | 0.008 | 0.0415 | 0.627 |
| *Summer grazing of winterage* |  | *274* | *113* | *41%* |  | *0.074* |  |  |
|  | No | 246 | 97 | 39% | Ref. |  |  |  |
|  | Yes | 28 | 16 | 57% | 2.048 | 0.076 | 0.929 | 4.517 |
| *Grazing commonage* |  | *274* | *113* | *41.24%* |  | *0.227* |  |  |
|  | Don't rent/own commonage | 225 | 95 | 42.22% | Ref. |  |  |  |
|  | Graze with 2 or 3 other herds | 16 | 7 | 43.75% | 0.062 | 0.905 | -0.961 | 1.084 |
|  | Graze with 4 or more other herds | 9 | 3 | 33.33% | -0.379 | 0.598 | -1.791 | 1.031 |
|  | Graze with 1 other herd | 14 | 7 | 50.00% | 0.313 | 0.569 | -0.766 | 1.394 |
|  | Own/rent but don’t graze it | 10 | 1 | 10.00% | -1.088 | 0.076 | -3.966 | 0.199 |
| *Cattle on winterage* |  | *271* | *113* | *41.70%* |  | *0.203* |  |  |
|  | None | 77 | 27 | 35.06% | Ref. |  |  |  |
|  | 1 to 25 | 111 | 42 | 37.84% | 1.127 | 0.698 | 0.615 | 2.064 |
|  | 26 to 50 | 58 | 31 | 53.45% | 2.126 | 0.034 | 1.059 | 4.267 |
|  | 51 to 75 | 15 | 7 | 46.67% | 1.62 | 0.397 | 0.531 | 4.952 |
|  | 76 to 100 | 10 | 3 | 30.00% | 0.793 | 0.752 | 0.189 | 3.320 |
|  | Over 100 | 3 | 3 | 100.00% | NA |  |  |  |

**Table S5:** Variables on nutrition and water associated with recent history of bTB breakdown on farm as reported from a survey of Burren farmers.

| Variable | Categories | N | N+ | % + | OR | P | Lower 95%CI | Upper 95%CI |
| --- | --- | --- | --- | --- | --- | --- | --- | --- |
| *Silage Feeding* |  | *272* | *112* | *41.18%* |  | *0.496* |  |  |
|  | Don’t use winterage | 78 | 29 | 37.18% | Ref. |  |  |  |
|  | Feed silage | 33 | 16 | 48.48% | 1.59 | 0.667 | 0.698 | 3.621 |
|  | Never feed silage | 123 | 54 | 43.90% | 1.322 | 0.392 | 0.739 | 2.364 |
|  | Only feed silage in bad weather | 38 | 13 | 34.21% | 0.878 | 0.364 | 0.389 | 1.979 |
| *Concentrate Feeding* |  | *272* | *112* | *41.18%* |  | *0.948* |  |  |
|  | Feed for 1 month | 34 | 14 | 41.18% | Ref. |  |  |  |
|  | Feed for 2 months | 56 | 23 | 41.07% | 0.996 | 0.992 | 0.418 | 2.367 |
|  | Feed for 3 to 4 months | 38 | 18 | 47.37% | 1.285 | 0.598 | 0.505 | 3.271 |
|  | Entire winter period | 5 | 2 | 40.00% | 0.952 | 0.96 | 0.14 | 6.464 |
|  | Don’t feed concentrates | 61 | 26 | 42.62% | 1.061 | 0.891 | 0.543 | 2.485 |
|  | Don’t use winterage | 78 | 29 | 37.18% | 0.845 | 0.689 | 0.371 | 1.925 |
| *Method of meal feeding* |  | *272* | *112* | *41.18%* |  | *0.735* |  |  |
|  | Directly on ground | 48 | 20 | 41.67% | Ref. |  |  |  |
|  | Don’t feed concentrates | 54 | 23 | 42.59% | 1.038 | 0.925 | 0.473 | 2.283 |
|  | Don’t use winterage | 78 | 29 | 37.18% | 0.828 | 0.616 | 0.397 | 1.728 |
|  | In a low trough | 10 | 6 | 60.00% | 2.1 | 0.295 | 0.523 | 8.424 |
|  | in a raised trough | 82 | 34 | 41.46% | 0.991 | 0.982 | 0.481 | 2.042 |
| *Water supply* |  | *271* | *112* | *41.33%* |  | *0.335* |  |  |
|  | Rock pools & other sources | 54 | 24 | 44.44% | Ref. |  |  |  |
|  | Directly from streams | 33 | 11 | 33.33% | 0.625 | 0.307 | 0.254 | 1.538 |
|  | From high troughs | 91 | 34 | 37.36% | 0.745 | 0.401 | 0.376 | 1.478 |
|  | From low troughs | 30 | 10 | 33.33% | 0.625 | 0.322 | 0.246 | 1.583 |
|  | Open well/spring | 20 | 11 | 55.00% | 1.527 | 0.421 | 0.544 | 4.286 |
|  | Biosecure source | 43 | 22 | 51.16% | 1.309 | 0.511 | 0.586 | 2.924 |
| *Trace elements* |  | *271* | *112* | 41.33% |  | *0.474* |  |  |
|  | Do not supplement | 45 | 22 | 48.89% | Ref. |  |  |  |
|  | Supplement with one product | 166 | 64 | 38.55% | 0.656 | 0.212 | 0.338 | 1.273 |
|  | Supplement with two products | 45 | 21 | 46.67% | 0.915 | 0.833 | 0.400 | 2.092 |
|  | Supplement with three products | 15 | 5 | 33.33% | 0.523 | 0.298 | 0.154 | 1.775 |

**Table S6:** Comparison of the top 10 ranked models based on Akaike’s Information Criterion (AICc) values.

| Rank | Diff. AIC | Badger sett accessibility | |  |  | Herd size |  |  |  | Dairy |  | Summer winterage grazing |  | Winterage time (Months) | | | |  |  |
| --- | --- | --- | --- | --- | --- | --- | --- | --- | --- | --- | --- | --- | --- | --- | --- | --- | --- | --- | --- |
|  |  | **<10%** | **11-30%** | **31%-50%** | **>50%** | **Small** | **Medium** | **Large** | **Very large** | **No** | **Yes** | **No** | **Yes** | **1** | **2** | **3** | **4** | | **0** |
| r1 | 0.000 | ref | 1.377 | 3.931 | 19.706 | ref | 1.401 | 2.172 | 3.084 | . | . | . | . | . | . | . | . | | . |
| r2 | 0.863 | ref | 1.364 | 4.054 | 19.232 | ref | 1.377 | 2.040 | 2.738 | ref | 1.672 | . | . | . | . | . | . | | . |
| r3 | 1.039 | ref | 1.358 | 3.810 | 18.211 | ref | 1.419 | 2.115 | 3.090 | . | . | ref | 1.543 | . | . | . | . | | . |
| r4 | 1.758 | ref | 1.344 | 3.931 | 17.711 | ref | 1.394 | 1.974 | 2.725 | ref | 1.730 | ref | 1.596 | . | . | . | . | | . |
| r5 | 2.709 | ref | 1.295 | 3.864 | 19.886 | . | . | . | . | ref | 2.450 | . | . | . | . | . | . | | . |
| r6 | 2.750 | ref | 1.367 | 3.948 | 14.222 | ref | 1.467 | 2.221 | 3.122 | . | . | . | . | ref | 0.218 | 0.307 | 0.223 | | 0.236 |
| r7 | 3.187 | ref | 1.278 | 3.741 | 18.125 | ref | . | . | . | ref | 2.522 | ref | 1.707 | . | . | . | . | | . |
| r8 | 4.108 | ref | 1.348 | 4.039 | 14.138 | ref | 1.436 | 2.104 | 2.837 | ref | 1.495 | . | . | ref | 0.235 | 0.334 | 0.247 | | 0.247 |
| r9 | 4.585 | ref | 1.302 | 3.569 | 21.415 | . | . | . | . | . | . | . | . | . | . | . | . | | . |
| r10 | 4.587 | ref | 1.362 | 3.915 | 14.092 | ref | 1.468 | 2.204 | 3.129 | . | . | ref | 1.219 | ref | 0.237 | 0.338 | 0.241 | | 0.261 |
